# Characterization and Bio-Accessibility Evaluation of Olive Leaf Extract-Enriched “Taralli”

**DOI:** 10.3390/foods9091268

**Published:** 2020-09-10

**Authors:** Annamaria Cedola, Carmen Palermo, Diego Centonze, Matteo Alessandro Del Nobile, Amalia Conte

**Affiliations:** Department of Agricultural Sciences, Food and Environment, University of Foggia, Via Napoli, 25, 71122 Foggia, Italy; annamaria.cedola@unifg.it (A.C.); carmen.palermo@unifg.it (C.P.); diego.centonze@unifg.it (D.C.); amalia.conte@unifg.it (A.C.)

**Keywords:** by-products, olive leaves, “taralli”, polyphenols, bio-accessibility, sustainable food

## Abstract

Olive leaves are rich in many compounds precious for human health. Due to this property, the current study was aimed to valorize the extract from this by-product in a cereal-based food, very popular all around the world, the “taralli”. To this aim, ultrasound-assisted extraction was applied to dried olive leaves to obtain the extract, used as “taralli” ingredient, instead of white wine. The “taralli” with and without extract was subjected to in vitro digestion to assess the quantity of polyphenolic compounds released in the gastrointestinal tract to become available for absorption. Total content of phenols and flavonoids, as well as the antioxidant capacity, was measured on both cooked and uncooked samples, before and after digestion. In addition, High-Performance Liquid Chromatography with Diode-Array Detection (HPLC-DAD) of the three most abundant polyphenols present in olive leaf extracts, such as oleuropein, hydroxytyrosol, and verbascoside, was carried out at the three stages of the digestion process. The results showed that the substitution of white wine with olive leaf extract increased the total content of polyphenols and flavonoids and the antioxidant capacity. Bio-accessibility of the main phenolic compounds demonstrated that oleuropein resisted slightly after gastric digestion but was almost completely degraded in the intestinal phase, while hydroxytyrosol and verbascoside were not resistant to the digestion process from the gastric phase.

## 1. Introduction

Olive leaves are agricultural residues resulting from the pruning of olive trees, as well as from olive mill leavings. These by-products are around 10% of the total weight of olives arriving to mills [1]. As reported in Silva et al. [2], olive leaves contain considerable amounts of phenolic compounds that generally attract remarkable interest in both food and pharmaceutical industry for the properties they possess. The concentration of bioactive compounds varies depending on many factors, such as olive origin and variety and product quality, but it is generally accepted that olive leaves can be used not only to obtain compounds with antimicrobial and antioxidant power but also for numerous medical treatments [3,4]. Benavente-Garcìa et al. [5] identified the main phenolic compounds present in the olive leaf extract obtained from *Olea europaea* L. of five cultivars, Villalonga, Alfafarenca, Picual, Cornicabra, and Blanqueta, from two south Spain regions (Andalucia and Murcia) and delineated the different antioxidant properties of these active compounds. Oleuropein, hydroxytyrosol, and verbascoside are recognized as the most abundant polyphenols identified in olive leaf extract [6]. It was demonstrated that these compounds and, in particular, oleuropein possess antioxidative, antimicrobial, antiviral, even against the HIV virus, anti-atherogenic, cardioprotective, antihypertensive, and anti-inflammatory properties. Furthermore, oleuropein has hypocholesterolemic and hypoglycemic activities [2,5,7]. In the overview of Wang et al. [8], the correlation between dietary polyphenols and obesity is well-recognized. Literature reports that also olive leaves, due to their important secondary metabolites with high free radical scavenging properties, can improve lipid metabolism to alleviate obesity problems, and various studies also support the role of olive polyphenols in the prevention of noncommunicable chronic diseases [9,10,11].

For the extraction of phenolic compounds from olive oil industrial by-products, both traditional and novel extraction methods were proposed [12]. As regards the solvent extraction, various biopolymers have been used as adsorbents for the recovery of polyphenols. Interesting results in terms of purification of oleuropein and rutin from olive leaf extracts were recorded with skin fibroin [13]. On the other hand, among the unconventional techniques, pressurized liquid extraction, microwave-assisted extraction, supercritical fluid extraction, and ultrasonic-assisted extraction can be cited [14,15,16,17]. Among them, ultrasound-assisted extraction is considered very interesting to extract compounds from vegetal materials [18]. It consists of equipment that is less expensive, much easier, and more efficient than other extraction methods. Thanks to these features, it can be also considered for possible use at the industrial level [12,19].

The applications of olive leaf extract in the food sector can be counted both as ingredient for food fortification and as active compound for food packaging applications. As regards food enrichment, two recent publications can be cited, dealing with cheese and beer, both enriched with olive leaf extract. Specifically, Noori et al. [20] studied the effect of olive leaf extract as prebiotic for survival of *Lactobacillus casei* in cheese during cold storage. The number of *L. casei* cells increased as the olive leaf extract added to cheese increased. It is interesting to observe that the extract addition did not affect cheese sensory quality; on the contrary, the product was more appreciated. Guglielmotti et al. [21] used olive leaves as beer ingredient. These authors confirmed that the addition of olive leaves highly increased polyphenol content and antioxidant capacity of beer, even though sensory analysis revealed that high concentration of olive leaves in the beer imparted an herbal aroma and a bitter and astringent taste. To face the problem caused by addition of by-products to food, the literature proposes microencapsulation as a promising technique to mask the unpleasant flavors [22]. Paulo and Santos [23], in their overview dealing with deriving valorization of phenolic compounds from olive oil by-products, highlighted the advantages of microencapsulation to protect the nutritional compounds in the enriched foods. In this context, also Flammini et al. [24] loaded microspheres with olive leaf extract by using the emulsification-internal gelation as microencapsulation method. The authors used alginate alone or in combination with pectin, whey proteins, or sodium caseinate. The presence of the polymers in the alginate-based microspheres increased the encapsulation efficiency, even though the pH of the system highly contributed to microspheres swelling and to the release rate of trapped phenolic compounds. The promising antimicrobial and antioxidant action of phenols present in fruit and vegetable by-products encouraged many researchers to integrate by-products in the form of powder or extract in plastic polymers for producing active films [25]. In this context, Cejudo Bastante and colleagues [26] produced active films with olive leaf extract and then tested the efficacy of the active films on cherry tomato. The antimicrobial capacity of the active systems was studied against the main, altering microorganisms, *Staphylococcus aureus*, *Escherichia coli*, *Salmonella enteritidis,* and *Pseudomonas aeruginosa*. The antimicrobial capacity of the impregnated films was very high in the case of *P. aeruginosa* and *S. enteritidis*, thus extending cherry tomatoes’ shelf life by 20 days.

A very few studies investigated the stability and the behavior of phenolic compounds after the digestion process. The bio-accessibility is the quantity of compounds released from the food matrix in the gastrointestinal tract, available for absorption [27]. Both in vitro and in vivo approaches can be used for the evaluation of the behavior of bioactive compounds during the digestion process. The in vivo method better simulates the digestion, even though this approach is complex, lengthy, and expensive [28]. On the contrary, models for in vitro digestion simulate the release of compounds from food matrix under gastrointestinal conditions [29], and in cheaper and simpler way permits recording simulation data [30].

Considering that numerous factors can affect the biochemical stability and the real accessibility of active compounds contained in the food matrix [6], the aim of this study was to develop a novel food, the “taralli”, enriched with olive leaf extract, rich in phenolic compounds. To the aim, white wine as traditional ingredient was substituted by olive leaf extract during “taralli” production. The total phenolic compounds, total flavonoids, and the antioxidant capacity were measured before and after cooking and before and after in vitro digestion to obtain information on the product’s nutritional content. Moreover, the sensory quality of enriched “taralli” was verified to assess the acceptability of this new fortified product. During in vitro digestion the degradation kinetics of three individual olive leaf polyphenols, oleuropein, hydroxytyrosol, and verbascoside, as the most abundant active compounds in olive leaf extract, were also assessed.

## 2. Materials and Methods

### 2.1. Raw Materials

The olive leaves (*Olea europaea,* Coratina cultivar) were dried in a dryer (SG600, Namad, Rome, Italy) at low temperature (35 °C for 48 h). Subsequently, a fine powder was obtained using a hammer mill (16/BV-Beccaria S.r.l., Cuneo, Italy). The powder was stored at 4 °C until it was used for the extraction.

### 2.2. Olive Leaf Extract

The olive leaf extract was obtained using an ultrasonic bath, model CP104 (39 kHz, 200 W), produced by C.E.I.A. S.p.A. (Viciomaggio, Arezzo, Italy), according to the method disclosed by the authors Sahin and Samli [31]. Briefly, 0.5 g of dried olive leaves and 10 mL of solvent (ethanol/water ratios 50:50, *v/v*) were used for the extraction at 25 °C for 60 min. The experiments were performed in triplicate and the resultant extracts were filtered through a 0.45-µm nylon syringe filter before use.

### 2.3. “Taralli” Preparation

“Taralli” was prepared according to a common recipe, using an unleavened dough of flour (Flour 00, 250 g) with salt (3.75 g), sugar (15 g), extra-virgin olive oil (75 g), instant yeast (4 g), and white wine (75 g), all purchased from a local market (Foggia, Italy). All the ingredients were mixed and then “taralli” was made by hand. These samples will be named as control “taralli” with wine (T-CTRL-W). The enriched experimental sample (T-EXT) was prepared using the same ingredients as for the T-CTRL-W sample, with olive leaf extract (75 g) in place of white wine. For cooking both types of “taralli”, an electric conventional oven (H2810, Hugin, Milan, Italy) was used. Cooking conditions in terms of temperature and time were: 200 °C for 12 min.

### 2.4. Sensory Analysis

For the sensory evaluation, aimed at assessing the liking of the samples, a 9-point scale was used, where 1 corresponded to “extremely dislike”, 9 to “extremely liking”, and 5 represented the threshold for product acceptability. To the aim, a group of eight trained panelists, members of the Food Science laboratory, was taken into account. The panel performed a sensory evaluation of the “taralli”, quantifying the acceptability in terms of color, odor, taste, aspect, and friability. The panel was also asked to give score for overall quality of “taralli”, on the basis of the above sensory attributes, quantifying the global judgement on the same scale. Panelists involved in the current study were people with several years of experience in sensory evaluation of food products; in any case, they were retrained for this study, in a 3-h session in which the product quality indices and the terminology were defined. During the sensory analysis, the samples were served in a random order, encoded with a three-digit code. To rinse the mouth between tastings, each panelist was provided with smooth water.

### 2.5. In Vitro Bio-Accessibility of Bioactive Compounds from Enriched “Taralli”

To assess the bio-accessibility of the nutritional compounds present in the enriched “taralli”, in vitro digestion was carried out as reported in the study of Cedola et al. [32]. The technique consists of the analysis of aliquots of samples from three different stages of digestion, the oral, the gastric, and the small intestine phases. Each sample, for both control and enriched “taralli”, was filtered with a 0.45-μm polytetrafluoroethylene (PTFE) filter and stored at 4 °C for subsequent analysis.

### 2.6. Total Phenolic Compounds, Total Flavonoids, and Antioxidant Activity

The extraction to determine total phenols, flavonoids, and antioxidant capacity was performed according to the study of Marinelli et al. [33]. Total phenolic compounds of both “taralli” sample and olive leaf extract were determined at 740 nm by UV-VIS spectrophotometer (UV1800, Shimadzu Italia Srl., Milan, Italy), using the Folin-Ciocalteu method [34], using a standard curve with different gallic acid concentrations (3.125–100 mg L^−1^, *y* = 109.43*x* + 4.4698, *R*^2^ = 0.999, Limit of Detection (LOD) = 2.5 mg GAE kg^−1^, Limit of Quantification (LOQ) = 7.6 mg GAE kg^−1^). The total phenol content (TPC) was expressed as milligrams of gallic acid equivalents (GAE) per gram of sample. Total flavonoid content was determined by aluminum chloride colorimetric method, as described by Chiung-Tsu et al. [35]. The calibration curve was made with standard solutions of quercetin (6.25–100 mg L^−1^, *y* = 502.93*x* − 4.8427, *R*^2^ = 0.999, LOD = 7.1 mg QE kg^−1^, LOQ = 21.4 mg QE kg^−1^) in order to express the total flavonoid content (TFC) as milligrams of quercetin equivalents (QE) per gram of dry sample weight (dw). The antioxidant capacity was assessed by using ferric-reducing ability of plasma (FRAP), as described by Mohd Salleh and Faraniza [36] with slight modifications. Briefly, 200 μL of “taralli” sample and leaf extract was mixed with 3 mL FRAP reagent and incubated in a water bath for 30 min at 37 °C. The absorbance was determined against blank at 593 nm. The FRAP values were calculated from a standard curve of different concentrations of FeSO4·7H2O (12.5 and 600 µM, *y* = 0.7306*x* + 0.0077, *R*^2^ = 0.999, LOD = 3 mmol Fe (III) kg^−1^, LOQ = 10 mmol Fe (III) kg^−1^). The antioxidant capacity was expressed as μmol of ferrous equivalent Fe (III)/g of dried sample. All tests were carried out in triplicate.

### 2.7. Identification and Quantification of Polyphenols by HPLC-DAD

To identify and quantify the main polyphenols in both control and enriched samples, a HPLC (Agilent LC 1100 series; Agilent Technologies, Inc., Palo Alto, CA, USA) was used. For the chromatographic separation of polyphenols, a reverse phase column, a Zorbax C18 5-μm (150 × 4.6 mm i.d., Agilent Technologies) column, was used. The elution was carried out using a linear gradient method with a solution of 2.5% acetic acid (A) and acetonitrile (B), starting the sequence with 10% B and programming the gradient to obtain 20% B at 10 min, 40% B at 35 min, 100% B at 40 min, and 100% B at 45 min. The following last 5 min were used to recondition the column with 10% B. The flow rate used was 1 mL/min, and the chromatograms were monitored at 240 (oleuropein), 280 (hydroxytyrosol), and 330 (verbascoside) nm. Compound identification was achieved by HPLC-DAD analysis with the help of pure standards and structural models already hypothesized in the literature [37,38]. Oleuropein, hydroxytyrosol, and verbascoside were quantified, using commercial standards, as the main olive leaf polyphenols. All the standards were purchased from Sigma Aldrich (Milan, Italy). The calibration curves were performed for the quantitative evaluation of the compounds, using, alternatively, oleuropein in ethanol and verbascoside and hydroxytyrosol in methanol, at five known concentrations suitable for the quantification of real samples. Polyphenol concentrations were expressed in mg of polyphenols per g of dry weight of olive leaves (g dw) or, in the case of standards, as mg of compound per mL of initial solution. The accuracy of the method for the determination of these polyphenolic compounds was assessed with recovery studies by adding standard compounds in triplicate blank samples (“taralli” without adding wine and polyphenol extract, therefore free of the analytes of interest). Nine concentration levels were used to test the linearity range of responses. The linear regression equation for each standard curve was obtained by plotting the amount of standard compound injected against the peak area. The linear regression analysis produced an *R*^2^ value of at least 0.9983 for the various compounds, thus demonstrating the validity of the analytical method.

### 2.8. Statistical Analysis

Experimental data were compared using a one-way analysis of variance (ANOVA) with software STATISTICA 7.1 for Windows (StatSoft, Inc., Tulsa, OK, USA). Duncan’s multiple range test, with the option of homogeneous groups (*p* < 0.05), was carried out to determine significant differences among samples.

## 3. Results and Discussion

### 3.1. “Taralli” Characterization

Novel “taralli” was produced with olive leaf extract in place of white wine and then analyzed for chemical and sensory properties. As can been inferred from data listed in Table 1, from the sensory point of view, the enriched product was found generally acceptable as the control sample. This result was not obvious because the addition of by-products in the form of extract or powder generally compromises the sensory quality of food and requests technological options to adjust the perceived defects [22,33]. Cedola et al. [39] tested a dry, olive paste flour coming from olive oil production process to enrich fish burger and also found that a preliminary hydration of the by-products with milk before addition to the rest of the formulation represented a valid technological option to enhance the sensory perception of the processed fish product and a valid shrewdness to reduce the concentration of bitter components. In the case under investigation, the slight statistical differences (*p* < 0.05) between control and fortified “taralli” found in terms of color and taste (7.44 ± 0.18 vs. to 8.13 ± 0.23) were most probably due to the dark color of the extract and its high content in polyphenols. It is well known that olive leaves contain phenols, such as oleuropein, which has a very bitter and spicy taste [36]. The enriched “taralli” showed lower friability than the conventional product but it still recorded an overall quality score well above the acceptability limit (score = 5).

The total phenolic compounds (mg gallic acid/g dw), the flavonoids (mg quercitin/g dw), and the antioxidant capacity (µmol FeSO_4_·7H_2_O/g dw) measured by the FRAP method of both uncooked and cooked “taralli” samples are shown in Table 2. Not surprisingly, the obtained results indicated that olive leaves have high phenol content that improves the nutritional quality of enriched food, going from 0.53 mg GAE/g dw to 0.72 mg GAE/g dw for the uncooked sample, and from 0.43 mg GAE/g dw to 0.61 mg GAE/g dw for the cooked one. A significant (*p* < 0.05) increase was recorded also in terms of total flavonoids because, while the control sample before and after cooking presented flavonoid amount accounting for 0.09 mg quercitin/g dw, in the fortified “taralli” the concentration was fourfold higher. Looking at data in the last column of Table 2, it is possible to infer that the enriched sample also showed an antioxidant capacity higher than the control sample. These findings confirmed what was reported in the literature, for both cooked and uncooked “taralli”. In fact, numerous phenolic components in olive leaves have strong radical-scavenging activity [3,4] and, for this reason, the experimental evidence recorded was not surprising. The other striking feature of data reported in the same table is related to the effects of the cooking process. By comparing the results of nutritional content of uncooked and cooked “taralli”, we can see that the cooking process did not affect the concentration of polyphenols, flavonoids, and antioxidant activity. Further, the better antioxidant capacity observed in the enriched sample compared to the control product was probably due to the efficient extraction of bound polyphenols. Marinelli et al. [40] observed a similar trend when they studied spaghetti enriched with red grape marc. In particular, the authors observed that significant increase in phenolic compounds and antioxidant capacity was recorded when by-products from wine processing were added to the formulation. It was striking to observe that cooking did not affect the nutritional quality of fortified pasta.

The improvement in nutritional properties of fortified samples was ascribed to the extract utilized in the formulation instead of wine. In order to better underline differences between these two ingredients, a direct comparison in terms of total polyphenols, total flavonoids, and antioxidant capacity between white wine and extract from olive leaves was also carried out. Data are shown in Table 3. Data reported in the table confirmed that the increase in nutritional content observed in the enriched “taralli” was due to the extract used as raw material in the formulation in place of white wine. Specifically, the extract total phenol content was 24.08 mg GAE/g dw with respect to the 4.97 mg GAE/g dw found in white wine, about fivefold higher. These results confirm data from literature. In fact, other authors also demonstrated that olive leaves are a potential source of phenolic compounds [41,42]. It is also worth noting that the quantity of flavonoids in the extract, 33.27 mg QE /g dw with respect to the sole 1.70 mg QE/g dw found in white wine, was in accordance to Abaza et al. [43], who also found flavonoids as the most widely distributed group of olive leaf polyphenols. It was not surprising that significant differences (*p* < 0.05) between control and enriched food were also found in terms of antioxidant activity. In particular, an antioxidant capacity value more than threefold higher was recorded for “taralli” containing the extract compared to the reference sample.

A direct comparison between the total phenols, flavonoids, and antioxidant capacity that one would expect in “taralli” samples based on the properties of crude extracts and the measured values was not provided due to the severe thermal treatment that “taralli” samples undergo. Besides, it must be considered that once the crude extract is mixed to the other “taralli” constituents to form a complex food matrix, a complete extraction of phenols and flavonoids is not possible anymore.

### 3.2. In Vitro Digestion of “Taralli”

Because the phytochemicals added to food are useful for human health, it is of fundamental importance that they are absorbed in the gastrointestinal tract. For a nutritional assessment, it is, therefore, appropriate to consider the bio-accessibility of nutrients, that is, to verify the fate of the compounds after digestion, as they can undergo changes or even degradation in the gastrointestinal digestion process. In the current study, to identify the stability of the bioactive polyphenols, in vitro digestion of both control and enriched “taralli” was carried out. Data of total phenols, flavonoids, and antioxidant activity, before and after digestion, are listed in Table 4. Two orders of information can be reached from data reported in the table. Specifically, from one hand, the amount of polyphenol compounds increased after the in vitro digestion in both T-CTRL-W and T-EXT samples, and, on the other side, T-EXT sample showed a significantly higher concentration of bio-accessible total polyphenols than the T-CTRL-W sample. While this latter evidence could be expected, the first one requests some explanations. A possible explication of the increase of polyphenol compounds after the in vitro digestion could be that given by authors in Gawlik-Dziki et al. [44], who also found that the enzymes used during the intestinal digestive process led to release of bound phenol acids, as well as amino acids, from wheat proteins contained in the flour used as ingredient to make the product. The amino acids can combine with Folin-Ciocalteu reagent, thus following polyphenol reaction [45]. In terms of flavonoids, T-EXT sample also showed higher concentration of bio-accessible compounds than the T-CTRL-W sample, specifically threefold higher. In addition, while a significant amount of bio-accessible flavonoids was observed in the digested enriched product (0.88 ± 0.44 mg QE/g dw), no flavonoids were detected in the digested control food. As regards the antioxidant activity, comparing data in Table 4, enriched “taralli” showed a value higher than the control: 4.86 ± 0.04 vs. 3.48 ± 0.05 µmol FeSO_4_·7H_2_O/g. In addition, the antioxidant capacity also increased after the in vitro digestion for both control and enriched samples. These findings are not surprising because literature also assessed a positive correlation between total phenol content and antioxidant capacity [46]. To this regard, two examples of bread fortified with by-products can be cited, the bread fortified with Chenopodium quinoa leaves’ powder [44] and bread fortified with 10% dry olive paste flour [32]. In both cases, data recorded from nutritional composition in terms of phenol content and antioxidant capacity demonstrated that total phenols can be considered the main responsible factor for antioxidant properties of fortified bread.

### 3.3. Degradation Kinetics of Specific Polyphenols During In Vitro Digestion by HPLC-DAD

**Figure 1:**
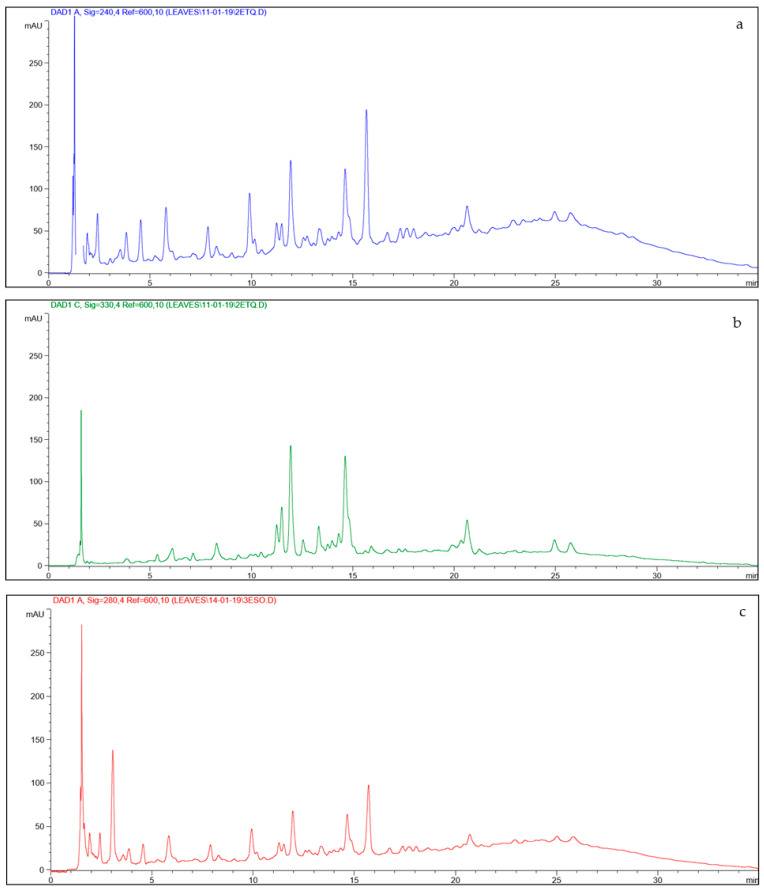
HPLC chromatograms of extract obtained from olive leaves. (**a**) Oleuropein, Retention time (min) = 15.73, λ = 240 nm; (**b**) Verbascoside, Retention time (min) = 11.90, λ = 330 nm; (**c**) Hydroxytyrosol, Retention time (min) = 3.09, λ = 280 nm.

As reported before, oleuropein is known to be the most abundant compound in olive leaves, while hydroxytyrosol is its precursor. Verbascoside is a conjugated hydroxytyrosol and caffeic glucoside [5,13]. It is known that only the polyphenols left in the gastrointestinal tract are really bioavailable in the intestine, but this does not apply to all the polyphenols present in the food matrix. To obtain information on the stability of the main polyphenols of olive leaves during digestion of “taralli”, the content of oleuropein, hydroxytyrosol, and verbascoside was monitored during the three different digestion phases (oral, gastric, and intestinal) by HPLC-DAD, and the results have been expressed as milligram of each polyphenol per g of dry “taralli” [40]. Typical HPLC-DAD chromatograms of samples extracted by ultrasonic bath are shown in Figure 1. The method of analysis showed very good recoveries, in the range 89–95%, and extended linear ranges of response (0.015–0.750 mg/g) that allowed an accurate determination of all the polyphenols investigated.

**Figure 2:**
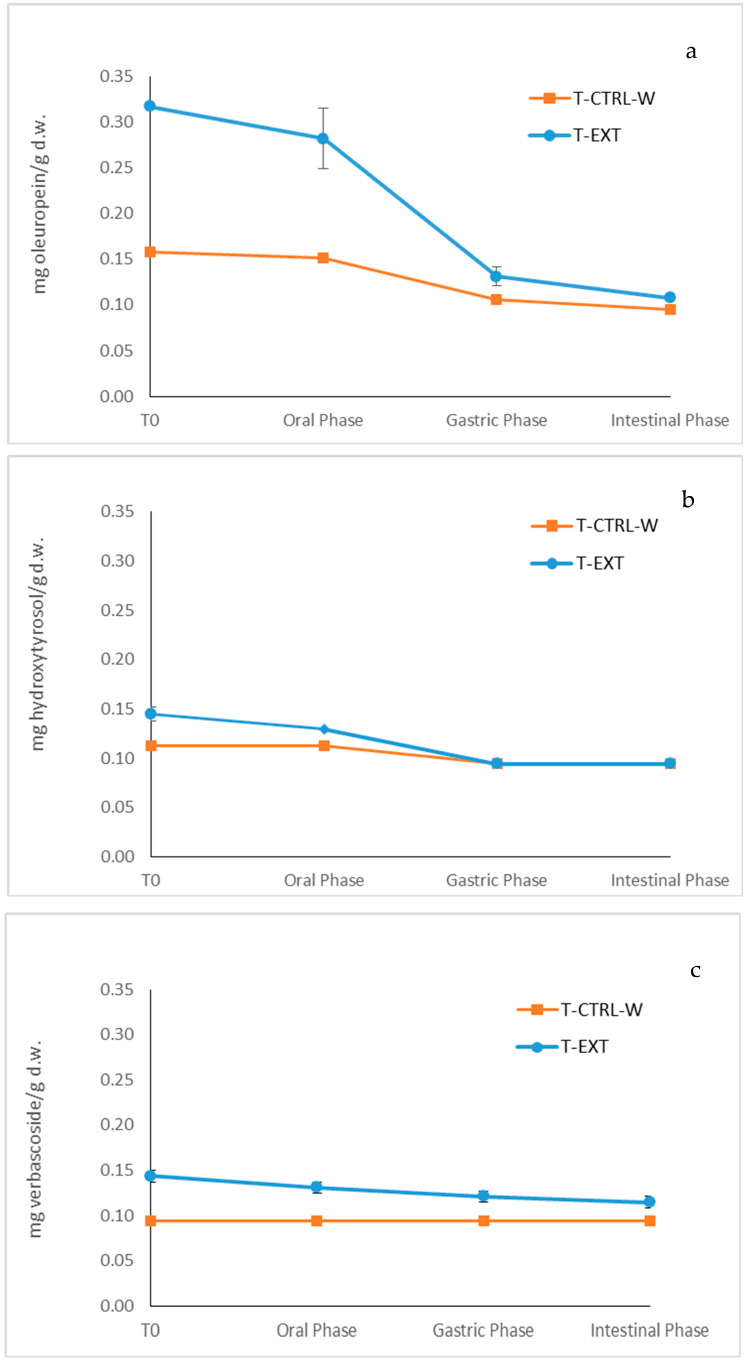
Degradation kinetics of oleuropein (**a**), hydroxytyrosol (**b**) and verbascoside (**c**) content during the in vitro digestion of “taralli”. T-CTRL-W: Control sample; T-EXT: “Taralli” with olive leaf extract.

Figure 2a–c presents the kinetics of the three compounds, giving values of the specific concentration (mg compound per g of dry weight) of each substance before digestion and after each step of the entire process, consisting of oral, gastric, and intestinal phases. As it can be seen from the Figure 2a, no significant decrease (*p* < 0.05) of the phenolic compound was observed during the oral phase, whereas the content showed a significant decrease (*p* < 0.05) in the gastric phase. This result suggests that the combined effect of enzymatic activity and pH changes helps to degrade part of the bioactive compound in the first phase of digestion. The decrease was more evident in the case of the oleuropein, being the most abundant among them, but some decrease can be also underlined for the other two compounds (Figure 2b,c). Oleuropein reached the duodenum but the intestinal phase influenced its quantity, so that oleuropein reduced its value to the same found in the control sample. Therefore, bio-accessibility of oleuropein was influenced by both pancreatic enzyme activity and alkaline pH. Ahmad-Qasem et al. [6], after HPLC-DAD/MS/MS analysis of olive leaves’ extract during digestion, also reported that oleuropein was abundantly decomposed in the intestinal phase.

Hydroxytyrosol and verbascoside presented a trend quite similar to that of oleuropein throughout the digestion (Figure 2b,c). More specifically, from the beginning of the process, hydroxytyrosol and verbascoside were much lower than the oleuropein value and, therefore, both control and enriched samples were found almost equal from the middle to the end of the digestion process. Not all the polyphenols behave the same way and their behavior can be influenced by several factors; only a fraction of phenols can be considered bio-accessible [47].

Tagliazucchi et al. [48] observed that the most abundant polyphenols in food are not necessarily more bio-accessible. This theory makes it clear that the differences in behavior could be also ascribed to the type of the matrix being digested. As a fact, in liquid matrices the polyphenols are readily bio-accessible, while, if the matrix is solid, the polyphenols contained must first be extracted to be bio-accessible and potentially bioavailable. The gastrointestinal tract is like an extractor in which both the mechanical action of chewing and the chemical action in the digestive phase operate. Both these phases contribute to the extraction of phenolic compounds from solid matrices. Therefore, the efficiency of polyphenol extraction can be affected but various factors in the gastrointestinal tract [49]. For example, studying the bio-accessibility of grape polyphenols, Tagliazucchi et al. [48] realized that the polyphenols released from grape during the first part of digestion caused their increase in this step.

## 4. Conclusions

In this study a potential valorization of olive oil by-products was proposed. In particular, the effects of olive leaf extract addition to “taralli” formulation, in place of white wine, was assessed and compared to control food in terms of sensory and nutritional properties. The significant difference between extract from olive leaves and white wine, recorded in terms of polyphenolic compounds, flavonoids, and antioxidant capacity, justified the interesting results also recorded for the enriched product, compared to control “taralli”. The novel product was as acceptable as the control “taralli”, thus suggesting that the extract addition improved product nutritional quality without affecting any sensory aspects. In addition, in vitro digestion was carried out on “taralli” to also assess its nutritional quality after consumption. The results demonstrate that the substitution of white wine with the olive leaf extract increased the total polyphenol, the flavonoid content, and the antioxidant activity. Finally, the analysis of the three common polyphenols of olive leaves was assessed to verify their bio-accessibility. The study reported in these pages has shown that, due to instability during the gastrointestinal digestion phase, the quantities of oleuropein, hydroxytyrosol, and verbascoside after digestion are almost negligible. Further study can be carried out to found other compounds fairly resistant to digestion that could be considered for the absorption process.

## Figures and Tables

**Table 1:** Sensory analysis of “taralli” samples.

Samples	Color	Odor	Taste	Aspect	Friability	Overall Quality
T-CTRL-W	8.13 ± 0.23 ^a^	8.44 ± 0.18 ^a^	8.13 ± 0.23 ^a^	8.25 ± 0.27 ^a^	7.88 ± 0.23 ^a^	8.25 ± 0.27 ^a^
T-EXT	7.44 ± 0.18 ^b^	7.75 ± 0.27 ^b^	7.44 ± 0.18 ^b^	7.75 ± 0.27 ^b^	7.44 ± 0.18 ^b^	7.88 ± 0.23 ^b^

Data in each column with different superscripts are significantly different (*p* < 0.05). T-CTRL-W: Control sample; T-EXT: “taralli” with leaf extract.

**Table 2:** Total content of phenols and flavonoids and antioxidant capacity of uncooked and cooked “taralli”.

	Total Phenols (mg GAE/g dw)		Total Flavonoids (mg QE/g dw)		Antioxidant Capacity (µmol FeSO_4_·7H_2_O/g dw)	
	Uncooked	Cooked	Uncooked	Cooked	Uncooked	Cooked
T-CTRL-W	0.53 ± 0.01 ^b^	0.43 ± 0.02 ^b^	0.09 ± 0.02 ^b^	0.09 ± 0.01 ^b^	2.07 ± 0.06 ^b^	3.48 ± 0.05 ^b^
T-EXT	0.72 ± 0.03 ^a^	0.61 ± 0.02 ^a^	0.39 ± 0.03 ^a^	0.36 ± 0.02 ^a^	3.10 ± 0.17 ^a^	4.86 ± 0.04 ^a^

Data in each column with different superscripts are significantly different (*p* < 0.05). T-CTRL-W: Control sample; T-EXT: “Taralli” with olive leaf extract.

**Table 3:** Phenols, flavonoids, and antioxidant capacity of wine and olive leaf extracts.

Sample	Total Phenols (mg GAE/mL)	Total Flavonoids (mg QE/mL)	Antioxidant Capacity (µmol FeSO_4_·7H_2_O/g dw)
White wine	4.97 ± 0.08 ^b^	1.70 ± 0.03 ^b^	140.58 ± 0.59 ^b^
Leaf Extract	24.08 ± 0.45 ^a^	33.27 ± 1.93 ^a^	518.17 ± 9.08 ^a^

Data in each column with different superscripts are significantly different (*p* < 0.05).

**Table 4:** Phenols, flavonoids, and antioxidant capacity of “taralli” samples before and after digestion.

Sample	Before Digestion			After Digestion		
	Total Phenols (mg GAE/g)	Total Flavonoids (mg QE/g)	Antioxidant Capacity (µmol FeSO_4_·7H_2_O/g)	Total Phenols (mg GAE/g)	Total Flavonoids (mg QE/g)	Antioxidant Capacity (µmol FeSO_4·_7H_2_O/g)
T-CTRL-W	0.39 ± 0.02 ^b^	0.12 ± 0.01 ^b^	3.48 ± 0.05 ^b^	2.23 ± 0.07 ^b^	n.i.	15.14 ± 0.19 ^b^
T-EXT	0.54 ± 0.04 ^a^	0.36 ± 0.02 ^a^	4.86 ± 0.04 ^a^	3.23 ± 0.17 ^a^	0.88 ± 0.04	20.98 ± 0.22 ^a^

Data in each column with different superscripts are significantly different (*p* < 0.05). T-CTRL-W: Control sample; T-EXT: “Taralli” with leaf extract. n.i. = not identified.
